# Small-Angle Neutron Scattering on Grain Boundaries of Cold-Rolled Nickel

**DOI:** 10.3390/ma19081608

**Published:** 2026-04-17

**Authors:** Tianfu Li, Zijun Wang, Wenyao Hu, Yiju Yang, Xiaoming Du, Shibo Yan, Zhong Chen, Taisen Zuo, He Cheng, Yongqin Chang, Dongfeng Chen

**Affiliations:** 1 China Institute of Atomic Energy, Beijing 102413, China; litianfu.li@163.com (T.L.); huwenyao@spic.com.cn (W.H.);; 2 College of Materials Science and Engineering, Shenyang Ligong University, Shenyang 110159, China; 3 China Spallation Neutron Source Science Center, Dongguan 523803, China; 4 Institute of High Energy Physics, Chinese Academy of Sciences, Beijing 100049, China; 5 School of Materials Science and Engineering, University of Science and Technology Beijing, Beijing 100083, China

**Keywords:** small-angle neutron scattering, low-q scattering, pure nickel, grain boundaries

## Abstract

The effect of grain boundaries on small-angle neutron scattering (SANS) was investigated for pure nickel. A series of annealed cold-rolled nickel samples were characterized by SANS, electron backscatter diffraction (EBSD), X-ray diffraction (XRD), and transmission electron microscopy (TEM). The experimental results indicate that the proportion of low-angle grain boundaries (LAGBs) has a noticeable influence on the scattering intensity. Cold-rolled samples exhibited a similar proportion of LAGBs, leading to only slight differences in scattering intensity. The scattering intensity was found to be dependent on annealing time and temperature. For samples with low degrees of recrystallization, a large number of LAGBs remained, resulting in high scattering intensity at low q. In contrast, samples with a high degree of recrystallization showed a significant reduction in LAGBs, which caused a noticeable decrease in SANS intensity.

## 1. Introduction

Small-angle neutron scattering (SANS) plays a pivotal role in unraveling the nanoscale heterogeneity inherent in materials. It has been widely employed to investigate perturbations in scattering length density (nuclear or magnetic), such as cavities, precipitates, dislocations, and magnetism in the majority of experiments [1]. It typically measures nanostructures within the size ranges of 1~100 nm, and serves as a powerful complementary technique to electron microscopy, atomic probes, and X-ray scattering. Neutrons are electrically neutral and endow SANS with the advantages of non-destructiveness and deep-penetrating capabilities, allowing for investigations of the internal structures of materials. Today, SANS has been extensively applied across diverse fields, such as biomedicine, aerospace, petrochemicals, and energy [2,3,4]. The unique properties of neutrons enable SANS to provide valuable insights into the intricate details of various materials, thereby significantly advancing research and development in these domains.

However, in SANS experiments, researchers have observed particularly strong scattering in the low-q region with q < 0.01 Å^−1^. This phenomenon may be related to grain boundaries, large-sized precipitates (e.g., carbides and oxides), or magnetic domain boundaries in magnetic materials [5,6,7]. The fundamental origin lies in the inhomogeneity of the scattering length density. Wang et al. investigated aged Fe-Cu model alloys and concluded that the rapid increase in scattering intensity in the low-q region was caused by grain boundaries and large-sized vacancy clusters [8]. However, for alloys, it is challenging to identify the exact contributing factor due to the presence of secondary phases and other defects at grain boundaries. Taglauer et al. studied deformed polycrystalline copper with a purity of 99.985% and found that scattering intensity was inversely proportional to grain size, implying a direct proportionality to grain surface density [9]. Thus, grain boundary diffraction may be the primary cause of low-q scattering.

Grain boundaries are a crystalline defect and exist widely in crystalline materials [10]. They have a significant impact on the material’s performance. Watanabe was the first to describe the distribution of various types and orientations of grain boundaries within materials by grain boundary character distribution (GBCD) [11]. According to the coincident site lattice (CSL) model, special grain boundaries disrupt random boundary connectivity, thereby improving the corrosion, creep and fracture resistance of the materials [12]. For decades, common approaches for manipulating the type and distribution of grain boundaries in materials have been realized through straining and annealing treatments [13,14,15].

Nickel or its alloys can form complex grain boundary structures, including grain orientation, defects, texture, and other features. After cold-rolling with large reduction and subsequent annealing treatment, these materials are well-suited as substrates for second-generation coated high-temperature superconductors. Ni substrate tapes with a strong cube texture, small boundary misorientation angles, uniform grain size, and a high-quality surface are required for the production of high-quality coated superconductors. Research into the recrystallization of highly cold-rolled nickel and the formation of low-angle grain boundaries is therefore important for both theoretical understanding and practical application.

To identify the low-q scattering caused by grain boundaries, we manipulate grain boundaries through cold-rolling and annealing treatments applied to the materials. After all, grain boundaries in polycrystals cause scattering primarily due to density inhomogeneity. Considering the potentially small density difference between grain boundaries and grains, as well as the presence of competing scattering phenomena—especially in alloys—by selecting high-purity nickel as the research material, the influence of secondary phases was effectively eliminated. In this work, we systematically investigated the low-q scattering behavior in deformed and annealed polycrystalline nickel.

## 2. Materials and Methods

### 2.1. Materials Preparation

For the as-received material, 99.99% high-purity nickel was employed. Sheets with dimensions of 15 mm × 15 mm × 1 mm were machined from the slabs and subjected to multi-step cross cold-rolling by a laboratory mill. Initially, all samples were subjected to an initial thickness reduction of 80%, followed by annealing at 700 °C for 10 min in a vacuum furnace to induce complete recrystallization, thereby enhancing the sample’s crystallinity and structural uniformity. Subsequently, thickness reductions of 20%, 40%, and 60% were applied to the samples, followed by annealing at 800 °C for the 20% reduction samples, with annealing durations ranging from 5 to 21 min. Annealing at 500–700 °C for 10 min was performed for the 20%, 40%, and 60% reduction samples. The cold-rolled sheets were named CR20, CR40, and CR60, respectively. For a comprehensive summary of the processing parameter combinations used in this work, refer to Table 1.

### 2.2. Microstructural Investigation

Phase identification and crystal structure analysis of the samples were performed using an X-ray diffractometer, Bruker AXS GmbH, Karlsrule, Germany, equipped with Cu-Kα, operated at 40 kV and 40 mA. The measurements employed a 2θ scanning range of 5°–90° and a scanning speed of 1°/min. Prior to EBSD analysis, the samples were subjected to argon ion polishing. An Oxford C-nano system, Oxford Instruments plc, Abingdon, Oxfordshire, United Kingdom was used to characterize the microstructural evolution of the samples, with data collected at a step size of 0.35~1.4 μm. Data processing was conducted using Channel 5 software. Low-angle grain boundaries (LAGBs) and high-angle grain boundaries (HAGBs) were defined as 2–15° and >15°, respectively. The samples were mechanically ground to a thickness of 50 μm and then thinned to a nanometer-scale thickness using ion milling to obtain high-resolution images. Once sample preparation was completed, the microstructure of the samples was characterized using a JEM-F200 field emission transmission electron microscope (TEM),JEOL Ltd., Akishima, Tokyo, Japan, with an acceleration voltage of 200 kV.

### 2.3. SANS

SANS experiments were performed using the Very Small Angle Neutron Scattering (VSANS) instrument BL-14, at the China Spallation Neutron Source, Dongguan, Guangdong, China [16,17]. Data reduction was carried out using the direct-beam method, which involved measurements of the direct beam, solid angle and transmission correction, as well as background subtraction. Neutrons with wavelengths in the range of 6–10.5 Å were utilized to achieve a simultaneous scattering vector range of 0.0016–0.6 Å^−1^, which probes mesoscopic length scales (>3900 Å). All measurements were conducted at room temperature. The diameter of the incident neutron beam was 8 mm and data acquisition lasted 60 min per specimen. The sample-to-detector distances were set to 4 m and 11.5 m. Scattering data reduction was carried out, including background reduction, transmission correction, detector efficiency calibration, and intensity normalization. The SANS intensity was obtained by inverse transformation of the experimental data [18]. It can be expressed as follows:

(1) $$I(q)=\Delta \eta^{2}\int_{0}^{\infty }N(R)V(R)F(q,R{)}^{2}dR+Bg$$

where ∆η is the difference in scattering length density between the scatters and the matrix; F(q, R) is the particle form factor; R is the scatter radius; V(R) is the scatter volume; N(R) is the log-normal size distribution function; and Bg is the background. For data analysis, the software SasView4.1 was used [19].

## 3. Results and Discussion

### 3.1. Microstructure

**Figure 1:**
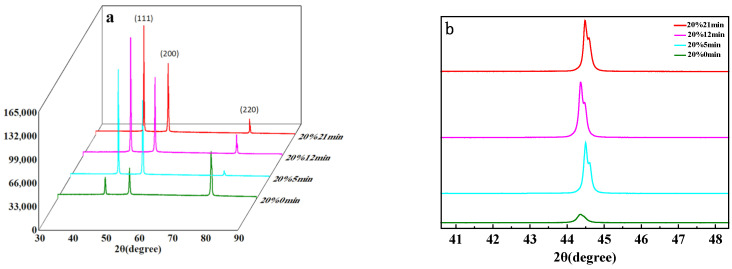
(**a**) Diffraction peaks of CR20 sample at different annealing times; (**b**) The (111) peaks of CR20 sample at different annealing times.

The XRD pattern of the 20% reduction samples is shown in Figure 1. There are only three diffraction peaks, (111), (200), and (220), at approximately 43°, 50°, and 75°, indicating that the peaks correspond to the face-centered cubic (FCC) structure of nickel [20]. As the annealing time increases, all peaks shift to higher 2θ angles, which can be attributed to the presence of non-uniform strain that causes structural changes [21]. After annealing, distorted lattices are recovered. In Figure 1b, the peaks of the CR20 samples broaden and split as the annealing time increases from 5 to 21 min. This phenomenon arises from the two closely spaced X-ray wavelengths emitted by the X-ray source, namely Kα1 and Kα2. The overlap of these two spectral lines results in peak splitting.

**Figure 2:**
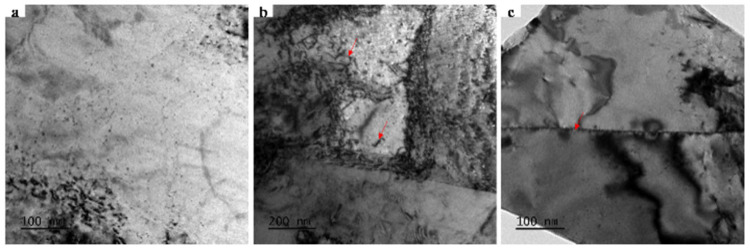
Microstructure of cold-rolled nickel: (**a**) Local morphology, (**b**) dislocations, (**c**) grain boundaries. The red arrows in (**b**) indicate dislocations. The red arrow in (**c**) indicates grain boundaries.

The TEM observations in Figure 2 reveal that the sample exhibits a uniform microstructure with no large precipitates detected. Only dislocations and grain boundaries are observed, indicating a relatively simple microstructure. In high-purity metal systems, the extremely low impurity content inhibits the formation of secondary phases during processing. Consequently, scattering signals originating from grain boundaries are more readily detectable.

### 3.2. Characterization of Grain Boundary

**Figure 3:**
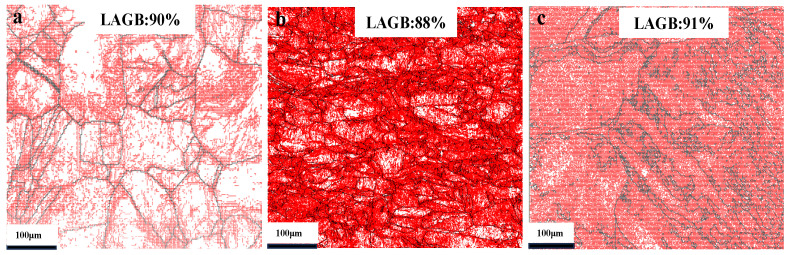
GBCD of cold-rolled samples: (**a**) 20%, (**b**) 40%, (**c**) 60% (LAGBs, red; HAGBs, black).

Figure 3 shows the electron backscatter diffraction (EBSD) maps of cold-rolled nickel, which clearly demonstrate the influence of cold-rolling on the grain boundary character distribution (GBCD). For the CR20 sample, the proportion of low-angle grain boundaries (LAGBs) is 90%. However, as the cold-rolling reduction increases, the proportion of LAGBs shows no significant change.

**Figure 4:**
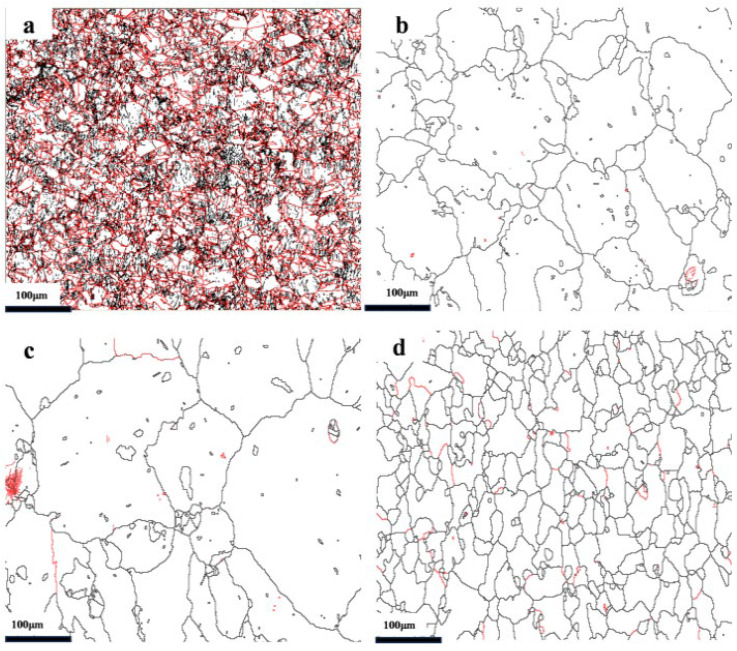
GBCD of CR20 at different annealing times: (**a**) 0 min, (**b**) 5 min, (**c**) 12 min, (**d**) 21 min. The red lines in the figures represent high-angle grain boundaries (HAGBs) with misorientation angles ≥ 15°, while black lines represent low-angle grain boundaries (LAGBs) with misorientation angles < 15°.

After 5 min annealing of the CR20 sample, the fraction of LAGBs decreases rapidly, as shown in Figure 4, and further annealing does not cause a significant reduction in the LAGB fraction. Compared with the grains before annealing, some grains exhibit significant growth after annealing for 5 and 12 min. Additionally, a large number of grains remain incompletely grown. When the annealing time is extended to 21 min, the grain size becomes more homogeneous, with no significant presence of large-sized grains. This indicates that during the high-temperature and short-time annealing process, some grains undergo rapid growth. When the annealing time reaches 21 min, the recrystallization degree is high.

In the process of recovery and recrystallization, dislocations undergo slip and climb, causing the polygonization of the cellular structure generated during plastic deformation, leading to the formation of subgrains. These subgrains gradually grow and develop into recrystallization nuclei, which absorb the surrounding distorted microstructure to form undistorted equiaxed grains, further reducing the dislocation density. The grain boundaries between these undistorted grains are predominantly composed of HAGBs.

**Figure 5:**
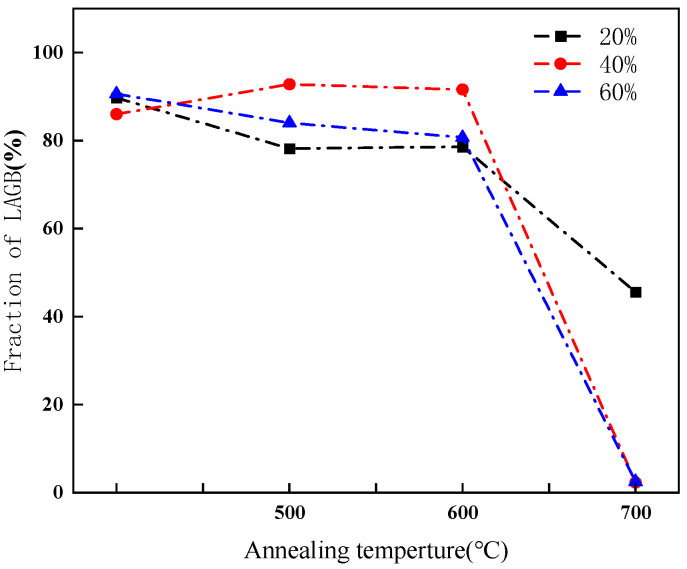
Fraction of LAGBs in the cold-rolled sample at different annealing temperatures.

The proportion of LAGBs in CR20, CR40 and CR60 samples annealed at various temperatures for 10 min is presented in Figure 5. At annealing temperatures of 600 °C or below, only a slight decrease in the proportion of LAGBs was observed. Interestingly, the CR40 sample exhibited a minor increase in LAGB proportion after annealing, which may be attributed to statistical errors in EBSD measurements. However, when the annealing temperature exceeded 600 °C, a significant reduction in the proportion of LAGBs was observed across all cold-rolled samples. Specifically, the proportion of LAGBs dropped to 2% in CR40 and CR60, whereas CR20 retained a relatively high LAGB proportion.

**Figure 6:**
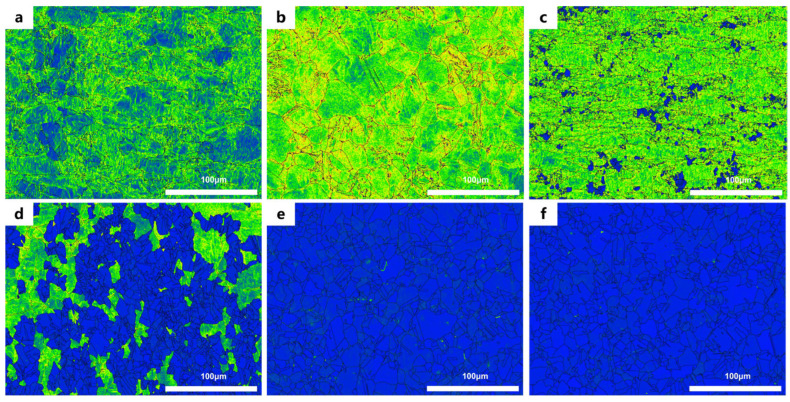
Local Average Misorientation (LAM) maps of the annealed sample: (**a**) 20%, (**b**) 40%, (**c**) 60% at 600 °C; (**d**) 20%, (**e**) 40%, (**f**) 60% at 700 °C. The color scale represents the local lattice misorientation, where blue indicates low misorientation (fully recrystallized regions) and green/yellow indicates moderate misorientation (partially recrystallized or deformed regions). The black lines denote grain boundaries.

LAGBs are composed of dislocations. Thus, based on the local average misorientation (LAM) map, a comprehensive explanation can be provided for the variations in LAGBs observed in Figure 6. When annealed at 600 °C, although recrystallization has occurred in the cold-rolled samples, there is no significant reduction in dislocation content, which results in a relatively high proportion of LAGBs. At an annealing temperature of 700 °C, CR20 still exhibits a high proportion of LAGBs due to incomplete recrystallization and the presence of significant high-density dislocations in some grains. In contrast, CR40 and CR60, which underwent higher degrees of cold-rolling, provide sufficient driving force for recrystallization, leading to a higher degree of recrystallization [11]. Consequently, the dislocation density decreases dramatically, resulting in a significant reduction in the proportion of LAGBs.

**Figure 7:**
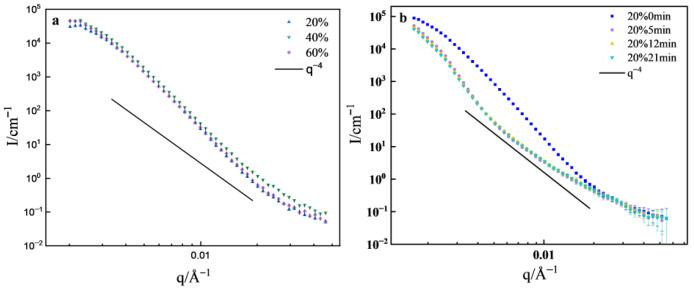
SANS curves: (**a**) cold-rolled sample, (**b**) CR20 annealed at different times.

The SANS curves for the cold-rolled samples are shown in Figure 7a. It can be seen that the scattering intensity of CR60 is almost the same as that of CR20, while CR40 exhibits a higher scattering intensity. As analyzed earlier, a large number of LAGBs form inside the cold-rolled samples, accompanied by increased unevenness in the matrix density, which leads to strong scattering in the low-q region. After the cold-rolling reduction reaches 20%, further increases in cold-rolling did not result in a significant change in LAGBs. Consequently, there is only a small difference in scattering intensity between CR20 and the other samples. The slopes of all cold-rolled samples at low-q are greater than 4. Schmidt pointed out that when the density of interfaces undergoes continuous changes, I(q) decreases more rapidly than a power law with exponent 4 [22]. The grain boundary serves as the transitional region between grains, and it is an interface with continuous density variation [23], resulting in a slope greater than 4 in the low-q region.

The SANS curves for CR20 under different annealing times are depicted in Figure 7b. In the range of q < 0.06 Å^−1^, the cold-rolled sample exhibits higher scattering intensity. After 5 min of annealing, the scattering intensity significantly decreases and exhibits a clear shoulder effect, indicating the presence of two distinct scatterers.

TEM observations reveal no large precipitates within the samples, so it might be the micropores, with scattering following q^−4^ in the high-q region (q > 0.006 Å^−1^). After annealing, there is a significant reduction in LAGBs, and micropores become the source of scattering signals in the high-q region. This is not relevant to our study and will not be further considered. After annealing for 12~21 min, the scattering intensity remains consistent with that after 5 min of annealing. According to electron backscatter diffraction (EBSD) results, CR20 initially has a higher density of LAGBs, which decreases rapidly within 5 min of annealing and reaches a relatively low level, with no significant changes during the subsequent annealing process.

Model-independent analysis is a mathematical method and the starting point for small-angle-scattering data analysis, which mainly includes analytical approaches such as Guinier and Porod analysis. Porod’s law mainly describes the variation in scattering intensity with the scattering vector. Under ideal conditions, for a two-phase system with constant scattering density and sharp interfaces, the following relation is satisfied at sufficiently large q [24]:

(2) $$\lim\left[q^{4}I(q)\right]=K$$

**Figure 8:**
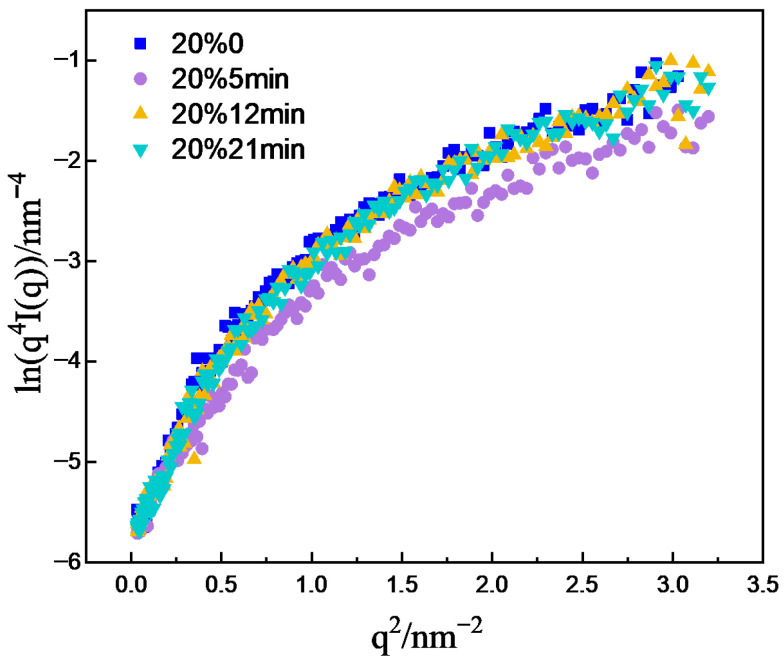
Porod curves of CR20 at different annealing times.

K is the Porod constant. However, owing to the complexity of real samples, both positive and negative deviations from Porod’s law can occur. For a quasi-two-phase system with sharp phase boundaries, fluctuations in scattering density within either phase give rise to excess scattering, resulting in a positive deviation from Porod’s law. In contrast, for quasi-two-phase systems with diffuse boundaries or an interfacial layer between the two phases, the scattering intensity is attenuated at high q, leading to a negative deviation from Porod’s law. The lnIq^4^-q^2^ plots of the one-dimensional scattering curves were subjected to Porod analysis, shown in Figure 8. The results revealed that in the high-q region, the curves did not converge to a constant value but exhibited a positive deviation. This suggests fluctuations in the scattering length density within the matrix. Although the density difference between the grain boundaries and grains may be small, when a large number of grain boundaries are present in the matrix, this difference can become significant. Therefore, the fluctuation is likely caused by the grain boundaries.

**Figure 9:**
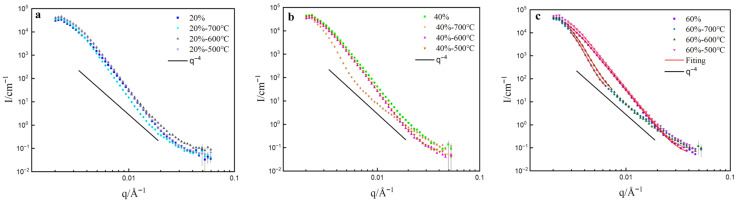
SANS curves of cold-rolled samples annealed at different temperatures: (**a**) 20%, (**b**) 40%, (**c**) 60%. To quantitatively analyze the relationship between LAGBs and low-q scattering, the scattering intensity at q = 0.004 Å^−1^ was taken for reference, as shown in Figure 10. It illustrates a linear correlation between scattering intensity and the proportion of LAGBs, indicating that scattering intensity increases proportionally with the increase in LAGBs.

The SANS curves of cold-rolling samples annealed at different temperatures are presented in Figure 9. At annealing temperatures of 600 °C and below, there is almost no difference in the low-q scattering curve of CR20. When the annealing temperature reaches 700 °C, a slight decrease in scattering intensity is observed, which is attributed to the reduction in LAGBs. For CR40, the scattering intensity of the cold-rolled sample is slightly higher than that of the annealed sample, due to its higher density of LAGBs. After annealing at 700 °C, a significant decrease in scattering intensity is observed, which correlates with the disappearance of LAGBs, as revealed by EBSD. The presence of numerous LAGBs leads to significant inhomogeneity in the matrix density. After annealing at 700 °C, recrystallization occurs, eliminating most defects and generating equiaxed, undistorted grains, resulting in a certain degree of uniformity recovery.

**Figure 10:**
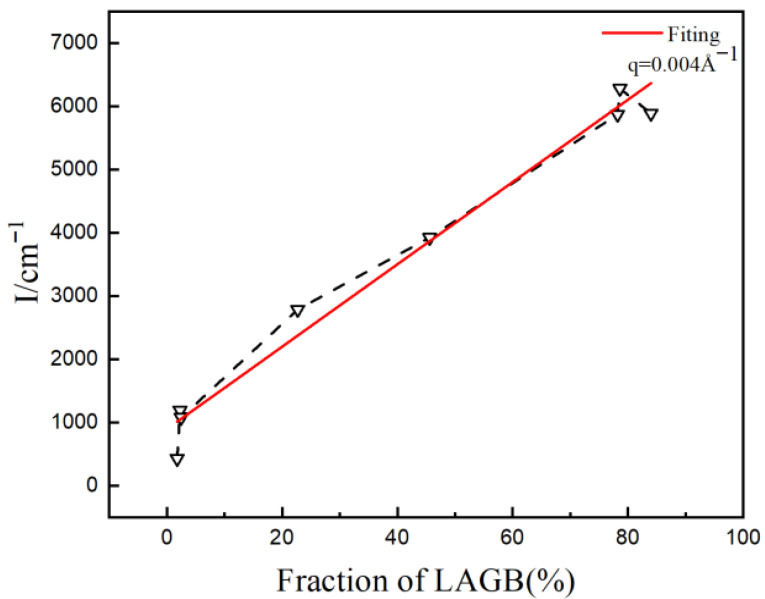
Relationship between scattering intensity and fraction of LAGBs.

In the case of CR60, despite annealing at 500 °C, a significant number of LAGBs remain due to severe cold-rolling deformation. Therefore, the scattering curve is similar to that of the cold-rolled sample. When annealed at 600 °C and 700 °C, a significant decrease in scattering intensity is observed. However, EBSD results show that a large number of LAGBs still exist after annealing at 600 °C. This appears contradictory to the observation that scattering intensity only decreases significantly when there is a substantial reduction in LAGBs. This discrepancy may be attributed to the fact that EBSD statistics only represent the distribution of grain boundaries on individual surfaces of the material, whereas SANS provides information about the bulk, offering better statistical representation. The scattering intensity after annealing at 700 °C only exhibits a slight decrease, indicating that the matrix density has become relatively uniform after annealing at 600 °C, and further increasing the annealing temperature does not result in significant changes—consistent with the results from different annealing times.

The radius of gyration (R_g_) and shape of the scatter were calculated from the 1D SANS patterns using Guinier–Porod model fitting [25]. The dimension variable reflects the geometric shape of the scatter: spherical (s = 0), rod (s = 1), and lamellae or disc (s = 2). The fitting results indicate that with increasing cold-rolling reduction, R_g_ gradually decreases, which may be attributed to compression deformation and grain fragmentation, resulting in a reduction in grain boundary size (Table 2). The value of s approaching 2 suggests that the morphology of the grain boundaries is approximately lamellar or plate-like. According to Long et al., this large-range small-angle scattering is caused by planar defects such as dislocation walls [26]. The EBSD observations in Figure 4 and Figure 6 reveal a large number of planar defects existing in the form of grain boundaries in the sample, which is consistent with the SANS analysis results.

When the cold-rolling reduction remains constant, the R_g_ gradually increases as the annealing temperature rises. Likewise, maintaining a constant annealing temperature while increasing the cold-rolling reduction results in a larger R_g_. This is likely due to the enhanced recrystallization process at higher temperatures and greater cold-rolling reduction, leading to faster recrystallization and larger R_g_ values. The consistency of the dimension variable s being close to 2 suggests that the morphology of the grain boundaries remains unaffected by the cold-rolling and annealing processes.

## 4. Conclusions

This study provides a comprehensive characterization of the microstructure of pure nickel using transmission electron microscopy (TEM), electron backscatter diffraction (EBSD), X-ray diffraction (XRD), and other methods. It reveals the relationship between grain boundary characteristic distributions and small-angle neutron scattering (SANS) signals in pure nickel under different processing conditions. This enables us to comprehensively characterize and deeply understand the low-q small-angle scattering behavior of grain boundaries, thereby providing theoretical and experimental guidance for SANS data analysis. The main conclusions are as follows:

(1) After annealing for 5 min, the proportion of LAGBs was reduced to 3.6%. When the annealing time is extended to 12~21 min, the proportion of LAGBs shows little change.
(2) When the annealing temperatures are above 600 °C, the LAGBs of all cold-rolled samples are significantly reduced. The proportion of LAGBs in the CR40 and CR60 cold-rolled samples was reduced to 2%, while there are still a large number of LAGBs in the CR20 cold-rolled samples.
(3) For CR20 annealed at 800 °C, and CR40 and CR60 annealed at 700 °C, the proportion of LAGBs is very low, and the scattering intensity shows a significant decrease. In contrast to other samples that contain a large number of LAGBs, the matrix presents substantial density heterogeneity, resulting in higher scattering intensity. After annealing, the significant disappearance of LAGBs leads to a pronounced decrease in scattering intensity.

## Figures and Tables

**Table 1:** Sample heat treatment information.

Reduction (%)	Annealing Time (Minute)	Annealing Temperature (°C)
20	0	800
	5	800
	12	800
	21	800
	10	500
	10	600
	10	700
40	10	500
	10	600
	10	700
60	10	500
	10	600
	10	700

**Table 2:** Fitting results of SANS data.

Sample	q Range/Å^−1^	Size (R_g_)/nm	s
20%	0.003–0.04	32.8 ± 0.1	1.8
40%	0.003–0.04	35.8 ± 0.2	1.7
60%	0.003–0.04	17.6 ± 0.2	2.4
20% 5 min (800 °C)	0.002–0.006	79.6 ± 0.3	1.8
20% 12 min (800 °C)	0.002–0.006	85.5 ± 0.4	1.7
20% 21 min (800 °C)	0.002–0.006	85.7 ± 0.4	1.7
20%-500 °C	0.003–0.04	30.8 ± 0.2	1.9
20%-600 °C	0.003–0.04	30.5 ± 0.7	1.9
20%-700 °C	0.003–0.04	36.7 ± 0.3	1.8
40%-500 °C	0.003–0.04	37.6 ± 0.2	1.7
40%-600 °C	0.003–0.04	37.1 ± 0.2	1.8
40%-700 °C	0.003–0.006	39.0 ± 0.1	1.7
60%-500 °C	0.003–0.04	51.6 ± 0.2	1.8
60%-600 °C	0.003–0.006	52.9 ± 0.4	1.6
60%-700 °C	0.003–0.006	53.9 ± 0.5	1.7

## Data Availability

The original contributions presented in this study are included in the article. Further inquiries can be directed to the corresponding authors.
